# Synbiotic Supplementation with Probiotics and Omega-3 Fatty Acids Enhances Upper-Body Muscle Strength in Elite Swimmers: Evidence for Gut–Muscle Axis Modulation During Race-Pace Training

**DOI:** 10.3390/nu17182959

**Published:** 2025-09-15

**Authors:** Babak Imanian, Mohammad Hemmatinafar, Ideh Maymandinejad, Mohammad Reza Binazade, Ralf Jäger, Zeinab Jahan, Kimia Naseri, Rasoul Rezaei, Katsuhiko Suzuki

**Affiliations:** 1 Department of Sport Science, Faculty of Education and Psychology, Shiraz University, Shiraz 71345, Iran; 2 Increnovo LLC, Whitefish Bay, WI 53217, USA; 3 Faculty of Sport Sciences, Waseda University, 2-579-15 Mikajima, Tokorozawa 359-1192, Japan

**Keywords:** gut–muscle axis, probiotics, omega-3 fatty acids, synbiotic supplementation, gut microbiota, athletic performance, muscle strength, ultra-short race-pace training

## Abstract

**Background:** The gut–muscle axis is believed to influence training adaptations through microbiota-derived signals and the regulation of inflammation, but evidence in elite swimmers is limited and mixed. This study aims to determine whether synbiotic supplementation (probiotics + omega-3) combined with ultra-short race-pace training (USRPT) improves sprint-related upper-body strength. **Methods:** In a randomized, double-blind, 8-week trial of male elite sprint freestyle swimmers, participants completed USRPT and were allocated to either synbiotic supplementation or its single-component arms (probiotic or omega-3) or placebo. Primary outcomes indexed dynamic/explosive strength (isokinetic shoulder torque and power at 180°/s, rate of force development, time-to-peak torque); secondary outcomes included maximal strength (MVIC; 60°/s) and field/strength-endurance tests (dead-hang, handgrip, medicine-ball throw). Analyses reported *p*-values with effect sizes. **Results:** The synbiotic group showed greater improvements in high-velocity, sprint-relevant measures versus comparators—higher 180°/s torque and power, increased rate of force development, and shorter time-to-peak torque (Time × Group *p* < 0.05 across domains; effects in the medium–large range). Changes in handgrip and medicine-ball throw were small and not different between groups (*p* > 0.05). **Conclusions:** Synbiotic supplementation concurrent with USRPT preferentially enhances dynamic (explosive) upper-body strength in elite sprint swimmers, whereas non-stroke-embedded field tests show limited added value. Any reference to gut–muscle-axis modulation is hypothesis-generating, as stool sequencing and metabolite profiling were not performed. Larger, sex-inclusive trials incorporating in-water, stroke-embedded assessments and microbiome/metabolomic profiling are warranted.

## 1. Introduction

Swimming has been part of the Olympic program since the inaugural modern Olympic Games and remains one of the most prominent disciplines, comprising 37 events that range from 50 to 10,000 m in distance [1]. Notably, 27—accounting for approximately 73%—of these events cover distances of 200 m or less, which are typically completed in under 2 min and 20 s [2]. Sprint-event performance in swimming depends on the capacity to generate high mechanical power and propulsive force in the water; consequently, upper-body strength is a primary determinant of swimming velocity [3]. In practice, coaches implement strength and conditioning (S&C) to target dynamic and, particularly in short events, explosive strength [4]. A 2023 review reported that most swimming strength and conditioning (S&C) programs rely on traditional resistance exercises and, less often, combine these with plyometric and/or core training. Across studies, these approaches were generally effective in improving performance [5]. In line with these demands, S&C—often delivered as dry-land training—is widely used to enhance sprint-relevant force and power capacities in competitive swimmers [4]. In competitive swimming, performance is defined by how quickly the prescribed distance is covered [6]. Previous research has shown that propulsive efficiency depends on the generation of high impulse forces against the water, with the upper-body musculature contributing the majority of force and velocity during swim strokes [7]. Consequently, a strong correlation exists between upper-body muscular strength and swimming performance [8]. To mitigate age- or training-related declines in muscle mass, strength, or physical performance, interventions such as physical training and nutritional supplementation—including protein, caffeine, probiotics, and omega-3 fatty acids—are frequently implemented [9].

Humans cannot synthesize the essential parent fatty acids—linoleic acid (*n*-6) and α-linolenic acid (*n*-3)—so they must be obtained from the diet (or supplements) [10]. Alpha-linolenic acid (ALA) undergoes a relatively inefficient enzymatic desaturation process, with a conversion rate of less than 1%, to form eicosapentaenoic acid (EPA) and docosahexaenoic acid (DHA) [11]. These long-chain fatty acids serve as precursors to eicosanoids such as prostaglandins, thromboxanes, and leukotrienes, which exhibit anti-inflammatory, antithrombotic, antiarrhythmic, and vasodilatory properties [12]. Consuming fish or fish oil provides EPA and DHA directly, bypassing the enzymatic competition required for ALA conversion [13]. Additionally, evidence suggests that EPA and DHA may help maintain or enhance muscle strength [14]. Meta-analyses indicate that increasing EPA and DHA intake can enhance omega-3 levels, promoting cardiovascular health and well-being [15]. One meta-analysis examining 10 studies found moderate evidence supporting the positive effect of *n*-3 polyunsaturated fatty acids (PUFAs) on muscle mass, particularly when supplementation exceeds 2 g/day; EPA and DHA appear to play a modest yet effective role in preserving or enhancing muscle strength [9]. Moreover, studies of co-encapsulated probiotic–omega-3 formulations suggest additive—sometimes synergistic—effects compared with either component alone [16].

The gut microbiota comprises microorganisms residing in the digestive tract, with an estimated population exceeding 10^14^ cells. Its collective genome is approximately 150 times larger than that of humans and around 10 times larger than a typical bacterial cell [17]. Gut microbial diversity and community composition are key determinants of intestinal homeostasis. Probiotics help maintain gut health and may improve nutrient bioavailability, including the absorption of protein-derived amino acids [18]. Research indicates that the composition and diversity of gut microbiota play a significant role in regulating skeletal muscle metabolism and function [19]. Findings from rodent models suggest that selected probiotics can mitigate sarcopenia/cachexia and influence performance-related pathways; however, these data are hypothesis-generating and not directly generalizable to elite swimmers [20]. Combining approaches to optimize muscle function—such as introducing specific microbes, tailored bacterial ecosystems, or microbial blends that adapt well to particular gut environments, alongside prebiotics and traditional anabolic supplements like proteins—may represent the most effective strategy for maintaining muscle health across all age groups [21,22]. The observed links between shifts in gut microbial communities, physiological dysfunction, and muscle catabolism suggest that the microbiota can influence muscle-mass regulation—directly or via systemic pathways. Probiotics, defined as live microorganisms that confer a health benefit at adequate intake, preserve or improve muscle mass and function in rodent models under both anabolic and catabolic conditions [23]. In athletes, the use of probiotics has been associated with improved overall health and, in some studies, modest gains in physical performance and exercise capacity [24]. Moreover, combining intensive training with probiotic supplementation has been reported to increase the relative abundance of lactic acid bacteria [25].

Training is the primary determinant of sprint swimming performance, and programs must be tailored to meet event demands. Ultra-short race-pace training (USRPT) breaks the race distance into brief intervals performed at race pace to target competition-specific physiology [26]. USRPT has been reported to lower heart rate, perceived exertion, and blood lactate, while improving speed, VO_2_max, strength, anaerobic capacity, and sprint performance [27,28]. Beyond these established benefits, there is growing recognition that high-intensity training modalities such as USRPT can also influence host physiology through changes in gut microbiota composition, metabolite production, and systemic inflammation [29,30]. These microbiome-mediated effects may interact with traditional performance adaptations, suggesting that comprehensive assessments of USRPT should not only encompass indicators such as explosive power, muscular endurance, agility, and sprint-specific indices but also consider their impact on gut microbiome diversity and function.

Probiotics and omega-3 fatty acids exert broadly comparable effects on immune regulation and intestinal barrier integrity [31]. Systematic reviews/meta-analyses indicate that each, when used alone, can lower inflammatory markers, such as IL-6 and CRP [32,33]. Because complementary mechanisms are plausible, combining them as a synbiotic may yield additive or synergistic benefits. Here, we use synbiotic to mean a product that pairs a probiotic (live microorganisms that confer a health benefit at adequate intake) with a prebiotic (a substrate selectively utilized by beneficial host microbes) to enhance probiotic survival and activity; in this context, long-chain omega-3 fatty acids can act as an “atypical prebiotic,” enriching favorable taxa and stabilizing the gut milieu to facilitate engraftment [34]. In mouse models of intestinal inflammation, co-administration of omega-3 (krill oil) with *L. reuteri*, alongside vitamin D, improved epithelial function by mitigating inflammation, supporting repair, and reducing pathogen-related injury [35]. Clinically, probiotics (VSL#3) and omega-3s each reduced hs-CRP, with the most significant decline observed when combined [36]. Evidence in athletes remains limited. In female collegiate swimmers, probiotic supplementation alone did not significantly alter performance or immune function during off-season training, despite shifts in microbiota composition [37]. Building on this foundation, Maymandinejad et al. reported that a synbiotic (probiotics + omega-3) delivered alongside USRPT in elite swimmers produced greater improvements in sprint performance, reaction time, and upper-body strength than either single agent or training alone [38], consistent with follow-up findings that synbiotics more effectively improve inflammatory/immune markers than single-component interventions in athletic cohorts [39].

Given the limited and sometimes conflicting evidence—and the still-emerging concept of the gut–muscle axis—it remains necessary to determine whether microbiome-targeted strategies can optimize training adaptations in elite swimmers. Probiotics and omega-3 fatty acids each modulate gut microbial composition, metabolite signaling, and systemic inflammation in ways that may influence skeletal muscle function. When combined as a synbiotic, they may synergistically enhance microbial diversity and function, thereby improving nutrient bioavailability, immune regulation, and recovery from exercise-induced stress. Because sprint events (50–200 m) depend mainly on upper-body explosive strength and rapid force production, we operationally distinguish among maximal strength (high force under isometric/low-velocity conditions), explosive/dynamic strength (rapid force generation reflected by rate of force development, time-to-peak torque, and power), and strength endurance (sustained force over time). USRPT may further interact with microbiome-mediated pathways by imposing substantial metabolic and inflammatory demands that remodel gut communities. This study is distinct from prior trials in its focus on elite sprint freestyle (front crawl) specialists, its concurrent implementation of a synbiotic (probiotics plus omega-3) within a USRPT framework, and its prioritization of dynamic/explosive upper-body strength outcomes (isokinetic 180°/s torque, average power, rate of force development, time-to-peak torque). Accordingly, our objective was to determine whether synbiotic supplementation alongside USRPT enhances upper-body muscular strength and power in competitive swimmers. Any reference to gut–muscle-axis modulation is hypothesis-generating, because the present study did not include stool sequencing or metabolite profiling. We hypothesized that, relative to controls, synbiotic plus USRPT would preferentially improve dynamic (explosive) upper-body strength—evidenced by higher isokinetic 180°/s torque, greater average power and rate of force development, and shorter time-to-peak torque—with complementary gains in maximal strength (MVIC; 60°/s torque) and strength endurance (dead-hang duration).

## 2. Methodology

### 2.1. Participants

Sixty male elite sprint freestyle (front crawl) swimmers (50–200 m) with ≥5 years of competitive experience, training ≥6 sessions·week^−1^ and with national-championship participation, were enrolled. To ensure homogeneous stroke-specific strength demands, only freestyle specialists were included in the study. Restricting enrollment to men minimized potential menstrual-cycle-related variability in neuromuscular and inflammatory markers to more accurately reflect the composition of the available elite sprint squad during recruitment. The inclusion of women with cycle-phase tracking is planned for subsequent trials. Anthropometric characteristics are shown in Table 1. Eligibility required professional status, absence of chronic disease/medical conditions, no known allergy to probiotics or omega-3 supplements, non-smoking status with alcohol abstinence, and no use of performance-enhancing drugs during the intervention. Participants abstained from smoking, alcohol, and caffeinated beverages for ≥24 h before each assessment (habitual caffeine intake outside this pre-test window was not restricted). All volunteers provided written informed consent and completed the PAR-Q. The study was approved by the Research Ethics Committee of the Faculty of Psychology and Educational Sciences, Shiraz University, Shiraz, Iran (IR.US.PSYEDU.REC.1403.045; 10 July 2024), and conducted in accordance with the Declaration of Helsinki.

### 2.2. Sample Size Calculation and Study Design

Sample-size planning was performed using G*Power 3.1.9.7 with the repeated-measures ANOVA option [40]. We specified an effect size of 0.25, α = 0.05, power = 0.80, the use of six groups and two time points, and the expected magnitude informed by prior work on probiotic effects on muscle performance, which reported moderate effects [41]. The computation yielded a minimum of 55 participants; allowing ≈for approximately 10% attrition, we enrolled 60 swimmers (10 per group).

**Figure 1:**
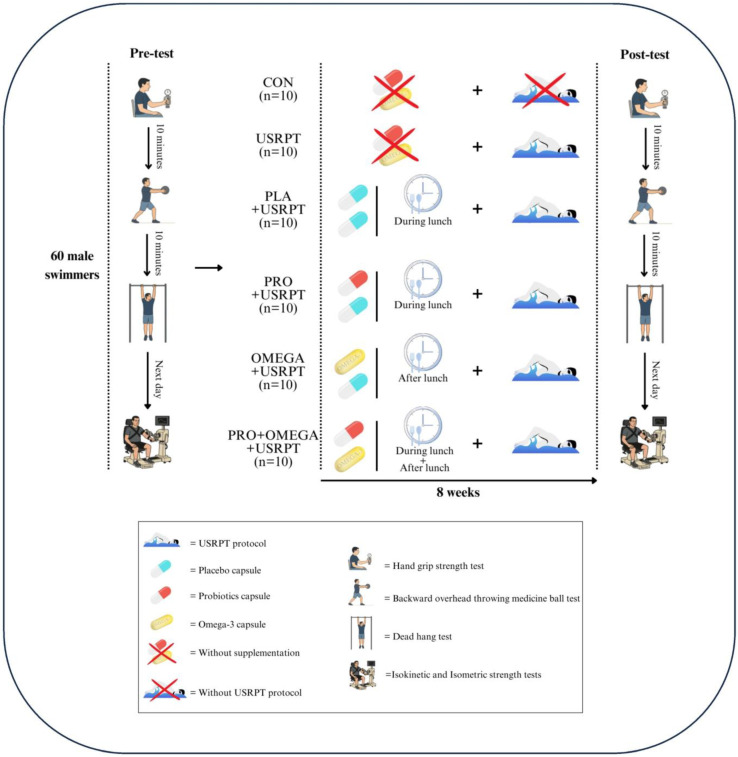
Study protocol and group allocation. Participants were randomized to six arms: Control (CON), USRPT, PLA + USRPT, PRO + USRPT, OMEGA + USRPT, and PRO + OMEGA + USRPT. Capsules for the supplement arms were identically packaged; color/shape differences in the schematic are for visualization only.

**Figure 2:**
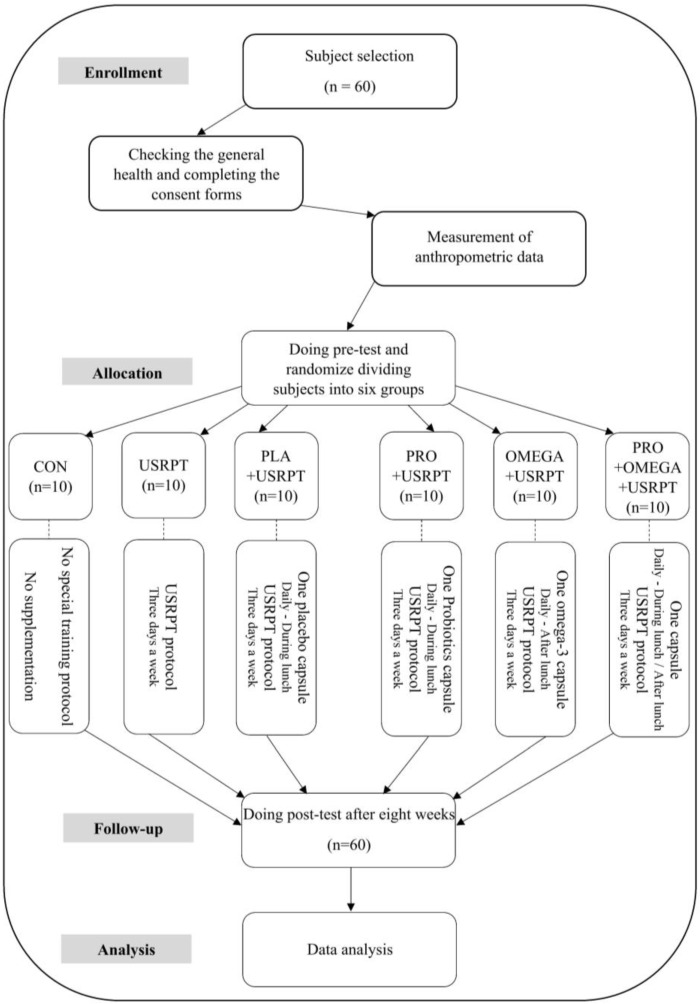
CONSORT flow diagram of participant enrollment, randomization, follow-up, and analysis.

The trial followed a randomized, double-blind, placebo-controlled design (Figure 1 and Figure 2). At screening, a licensed general practitioner confirmed eligibility and overall health status. After a full explanation of the aims, risks, and benefits, written informed consent was obtained. Participants then completed the PAR-Q and a standardized FFQ that captured information about their habitual diet, including fermented foods (e.g., yogurt, kefir, kimchi) and supplements (probiotics, prebiotics, omega-3 fatty acids) relevant to gut microbiota. During the 8-week intervention, athletes were instructed to maintain their usual dietary habits and to refrain from initiating the use of probiotic or omega-3 supplements. Intake of omega-3–rich fish and probiotic-rich foods was assessed at baseline and at week 8 via FFQ, complemented by a weekly checklist; deviations were recorded. At baseline, omega-3 exposure corresponded to ~1 oily-fish serving·week^−1^ (≈150–300 mg·day^−1^ EPA+DHA equivalent) with similar use of omega-6–rich cooking oils across groups; no between-group differences were detected (all *p* > 0.05). A familiarization visit introduced the testing apparatus, protocols, and pool environment (Shiraz University). To standardize conditions, participants refrained from vigorous activity and consumed no caffeinated products for at least 24 h before testing. Pre-test assessments were completed over two consecutive mornings (09:00–12:00). On day one, following a standardized warm-up, participants underwent functional strength assessments: dominant and non-dominant handgrip strength (DHGS and NDHGS), throwing-medicine-ball distance (TMB), and dead-hang tests with straight-elbow (SEDH) and flexed-elbow (FEDH) positions. On day two, isokinetic strength (shoulder extensors and flexors) was measured at angular velocities of 60°/s and 180°/s, and maximum voluntary isometric contraction (MVIC) was assessed at joint angles of 45° and 60°. A minimum active recovery period of 10 min was provided between tests to avoid fatigue. After baseline testing, participants were randomized via SNOSE into six arms (*n* = 10 each): Control (CON), USRPT only, PLA + USRPT, PRO + USRPT, OMEGA + USRPT, and PRO + OMEGA + USRPT. Allocation and supplement packaging were managed by personnel independent of data collection to preserve double-blinding; supplements were dispensed in identical containers. All USRPT groups completed a standardized eight-week program (3 sessions·week^−1^), and athletes consumed a standardized breakfast (250 kcal; 45 g carbohydrate, 9 g protein, 5 g fat) 90 min before each session [25]. Post-test measurements were obtained eight weeks after baseline under the exact timing and environmental conditions (09:00–12:00). Water was available ad libitum during all testing. All participants were trained within the same competitive program, with qualified coaches supervising throughout.

### 2.3. Blinding Procedure

Group assignment was concealed using the SNOSE procedure (sequentially numbered, opaque, sealed envelopes). An independent coordinator, not otherwise involved in the study, prepared sequential envelopes containing pre-specified allocation codes. Envelopes were issued in enrollment order and opened only after baseline testing was completed, so neither participants nor investigators knew the assignment before the intervention commenced. In parallel, supplements were coded and identically packaged by a separate third party, maintaining double-blinding for athletes, coaches, and assessors. These steps align with recommended standards for allocation concealment and bias reduction, thereby strengthening the internal validity [42].

### 2.4. Training Protocol

All participants completed an 8-week ultra-short race-pace training (USRPT) program integrated into the regular team schedule (3 sessions·week^−1^). USRPT prioritizes very short repeats at each swimmer’s event-specific race pace, brief rest intervals, and strict pace fidelity while preserving stroke quality under accumulating fatigue. Each supervised session followed the same structure: (i) standardized warm-up; (ii) a main set of race-pace repeats; and (iii) cool-down. The main set comprised 17 × 25 m repeats at individual race pace with 10 s rests, followed—after 5 min of active rest—by 17 × 12.5 m repeats at race pace with 5 s rests. The individual target pace for each repeat was derived from the swimmers’ most recent timed performances (e.g., 50 and/or 100 m), and coaches monitored split times in real-time using an electronic stopwatch (Stopwatch Selecta, W10710; Waterfly, Tokyo, Japan), providing immediate technical cues. If a swimmer failed to meet the target pace on two consecutive repeats, the rest time was adjusted or the set was terminated to preserve race-pace specificity and stroke mechanics. Progression across the 8 weeks was applied conservatively by adding two successful repeats per week when pace fidelity and technique were maintained. Training content (session structure, targets, and coaching feedback) was identical across groups; the only between-group distinction was the supplementation condition. Attendance and session RPEs were recorded at each session, and any adverse events were documented [2,26,43].

### 2.5. Supplementation Protocol

Supplementation spanned eight weeks (Figure 1 and Figure 2). All athletes ingested two capsules per day according to their allocation, with packaging and codes prepared to maintain blinding. The PRO + USRPT group received one probiotic capsule (Comflor PRO; Farabiotic Company, Tehran, Iran) containing eight strains totaling 4.5 × 10^11^ CFU (see Table 2) plus one placebo [18,44]. The OMEGA + USRPT group took one fish-oil capsule (EuRho Vital; Bönen, Germany) delivering 1000 mg fish oil (500 mg EPA, 180 mg DHA) together with one placebo [38]. The PRO + OMEGA + USRPT group consumed both actives each day (probiotic at lunch; omega-3 after lunch). The PLA + USRPT group received two starch capsules matched in external appearance to preserve masking. To reduce the chance of unblinding by taste or smell, EuRho Vital omega-3 capsules were odorless. Group allocations remained concealed throughout the trial. Adherence exceeded 95%, verified via weekly pill counts and daily intake logs, which is comparable to or better than the adherence reported in related exercise–nutrition trials [45].

## 3. Functional Tests

### 3.1. Handgrip Strength Test

Handgrip strength (HGS)—used as an index of upper-body isometric force and neuromuscular function—was assessed with a calibrated hydraulic hand dynamometer (Saehan SH5001; SAEHAN Corporation, Changwon, Republic of Korea; capacity 200 lbf/90 kg). Participants were seated with the elbow at 90° flexion; measurements were obtained for the dominant (DHGS) and non-dominant (NDHGS) hands. After standardized instructions, each swimmer performed three maximal isometric efforts per hand with a 1 min rest between trials; the highest value was retained for analysis. The testing order was randomized, and participants were instructed to discontinue if any pain occurred. Procedures followed established guidelines for isometric strength assessment in exercise science [46].

### 3.2. Backward Overhead Throwing-Medicine-Ball Test

Upper- and whole-body explosive power was assessed with the standing backward overhead medicine-ball throw (TMB), a task relevant to sprint starts, underwater phases, and rapid stroke acceleration. Participants stood on a marked line, feet shoulder-width apart, and facing away from the throw. Holding the ball with both hands, they executed a backward overhead swing and released the ball explosively behind the body. A slight countermovement was allowed, but both feet had to remain in contact with the floor throughout the attempt. Each swimmer completed three trials, and the greatest distance from the throwing line to the first point of ground contact was recorded for analysis [47,48]. The TMB test is a reliable measure of total-body explosive strength, emphasizing the posterior chain, core, and upper-body coordination. In sprint swimming, explosive upper-body and core power are critical for strong starts, turns, and stroke transitions, making this test a practical and valid field-based assessment of functional explosive power for competitive swimmers.

### 3.3. Dead-Hang Test

Upper-body isometric endurance was evaluated with two bar-hang variants. In the Straight-Elbow Dead-Hang (SEDH), swimmers grasped a horizontal pull-up bar with a shoulder-width pronated grip and elbows fully extended, maintaining the position until volitional fatigue or loss of position. In the Flexed-Elbow Dead-Hang (FEDH), they started with the chin above the bar and elbows flexed, holding the posture to the same termination criteria. Time-to-task failure (s) was recorded with a digital stopwatch; the best value was retained for analysis. A 3 min passive recovery separated the two variants. Notably, FEDH has demonstrated good reliability as a measure of upper-body isometric endurance in young athletes [49,50].

### 3.4. Isokinetic and Isometric Strength Tests

Isokinetic strength of the dominant shoulder extensors (EXT) and flexors (FLX) was assessed using a Biodex System 4 Pro dynamometer (Biodex Medical Systems, Shirley, NY, USA) under concentric conditions at 60°·s^−1^ and 180°·s^−1^. At each angular velocity, participants performed five maximal repetitions in an extension→flexion sequence, with a 60 s rest between sets. Gravity correction was applied using the manufacturer’s software. All tests were conducted in a seated position with the hip at 90°. For shoulder flexion (range of motion 0–140°), participant positioning followed the methods described by Wang et al. [51]. In contrast, shoulder extension (range of motion 0–130°) positioning followed the procedure described by Ekstrand et al. [52]. The following outcomes were obtained: absolute peak torque (APT), relative peak torque (RPT), time-to-peak torque (TPT), average rate of force development (AvRFD; APT/TPT), and average power (AvP). Assessments were performed at 60°/s and 180°/s to span distinct strength qualities. The 60°/s condition indexes maximal strength under controlled speed, characterizing peak torque capacity of the shoulder extensors and flexors. The 180°/s condition probes dynamic performance, reflecting the ability to generate rapid force and power in sport-relevant actions. Using both velocities yields a comprehensive strength–power profile and is consistent with established isokinetic testing practice [53,54].

Maximum voluntary isometric contraction (MVIC) of the dominant shoulder was assessed with the same dynamometer in extension and flexion at joint angles of 45° and 60°. At each angle, participants performed five maximal efforts of 5 s each with adequate rest between trials. The 45° and 60° positions represent mid-range angles where shoulder extensors and flexors typically approach near-peak torque due to advantageous length–tension characteristics; these angles are commonly used in shoulder MVIC protocols because they provide reliable measurements while limiting joint strain and enabling high force output [55,56]. Thus, they are well suited to detecting intervention-related changes in isometric strength.

To capture sprint-relevant dynamic upper-body strength, we prioritized high-velocity isokinetic shoulder rotations (180°/s), rate of force development, and time-to-peak torque, along with a medicine-ball throw, measures that are reliable in competitive swimmers and that show correlations with sprint performance [57].

### 3.5. Statistical Analyses

Data were summarized with descriptive statistics and analyzed inferentially. Normality was examined using the Kolmogorov–Smirnov test. Pre–post differences across the six groups were evaluated with a two-way repeated-measures ANOVA (Time [pre, post] × Group [six levels]). When main or interaction effects were significant, Bonferroni-adjusted pairwise comparisons identified specific contrasts. Within-group effect sizes were reported as Cohen’s *dz* from paired *t*-tests and interpreted as small (≥0.20), medium (≥0.50), large (≥0.80), and very large (≥1.00). For ANOVA effects, we reported the partial eta squared (ηp^2^), categorized as small (≥0.01), medium (≥0.059), and large (≥0.138) [58]. Results are presented as mean ± SD, with two-tailed significance set at *p* ≤ 0.05. Analyses were performed in SPSS v26 (IBM, Chicago, IL, USA), and figures were created in GraphPad Prism v9.0.0 (GraphPad Software, San Diego, CA, USA).

## 4. Results

Table 1 reports the participants’ anthropometric characteristics (mean ± SD). Table 3 summarizes, for all outcomes, the pre- and post-intervention means ± SD and the % relative change, including functional performance (DHGS, NDHGS, TMB, SEDH, FEDH), isokinetic shoulder strength parameters (APT, RPT, TPT, AvRFD, AvP for FLX and EXT at 60°/s and 180°/s), and isometric strength (MVIC at 45° and 60°).

### 4.1. Functional Tests

**Figure 3:**
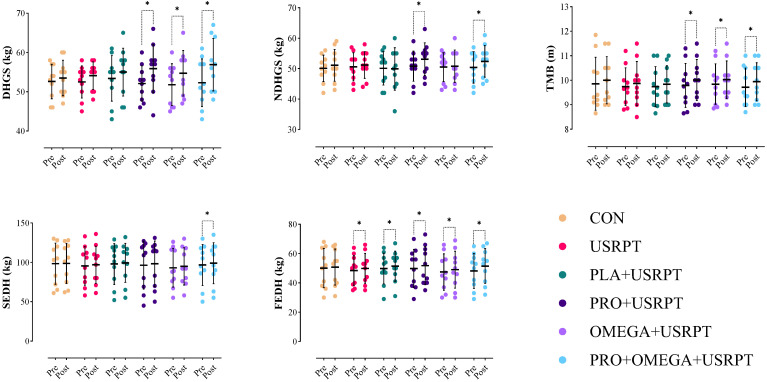
Means and standard deviations of the functional tests results in the six groups (control (CON), training (USRPT), placebo and training (PLA + USRPT), probiotics and training (PRO + USRPT), omega-3 and training (OMEGA + USRPT), and probiotics with omega-3 and training (PRO + OMEGA + USRPT)). DHGS: dominant handgrip strength; NDHGS: non-dominant handgrip strength; TMB: throwing medicine ball; SEDH: stretch elbow’s dead-hang; FEDH: flexed elbow’s dead-hang. *: Significant difference compared to the pre-test.

DHGS: A two-way repeated-measures analysis showed a significant main effect of Time (F_1.00_ = 54.091, *p* = 0.001, ηp^2^ = 0.500) and a significant Time × Group interaction (F_5.00_ = 2.850, *p* = 0.023, ηp^2^ = 0.209). Bonferroni-adjusted post hoc tests indicated pre–post increases in DHGS for PRO + USRPT (*p* = 0.001, *dz* = 1.618), OMEGA + USRPT (*p* = 0.001, *dz* = 1.033), and PRO + OMEGA + USRPT (*p* = 0.001, *dz* = 1.247), whereas CON, USRPT, and PLA + USRPT showed no significant change (all *p* > 0.05). Pre- and post-between-group comparisons were not significant (all *p* > 0.05); see Figure 3 and Table 4.

NDHGS: The two-way repeated-measures analysis revealed a significant Time effect on NDHGS (F_1.00_ = 8.995, *p* = 0.004, ηp^2^ = 0.143), with no Time × Group interaction (F_5.00_ = 1.433, *p* = 0.227, ηp^2^ = 0.117). Bonferroni-adjusted comparisons indicated pre–post gains in PRO + USRPT (*p* = 0.007, *dz* = 1.089) and PRO + OMEGA + USRPT (*p* = 0.018, *dz* = 0.594), whereas CON, USRPT, PLA + USRPT, and OMEGA + USRPT showed no significant change (all *p* > 0.05). Pre- and post-between-group differences were not significant (all *p* > 0.05; Figure 3, Table 4).

TMB: There was a significant main effect of Time (F_1.00_ = 20.630, *p* = 0.001, ηp^2^ = 0.276) but no Time × Group interaction (F_5.00_ = 0.326, *p* = 0.895, ηp^2^ = 0.029). Post hoc tests showed pre to post increases in PRO + USRPT (*p* = 0.018, *dz* = 2.250), OMEGA + USRPT (*p* = 0.041, *dz* = 0.708), and PRO + OMEGA + USRPT (*p* = 0.014, *dz* = 0.668), with CON, USRPT, and PLA + USRPT unchanged (all *p* > 0.05). Between-group comparisons at each time point were non-significant (all *p* > 0.05; Figure 3, Table 4).

SEDH: Analysis revealed a significant Time effect (F_1.00_ = 15.735, *p* = 0.001, ηp^2^ = 0.226) and a non-significant Time × Group term (F_5.00_ = 0.440, *p* = 0.819, ηp^2^ = 0.039). Only PRO + OMEGA + USRPT improved from pre- to post-intervention (*p* = 0.020, *dz* = 0.743); CON, USRPT, PLA + USRPT, PRO + USRPT, and OMEGA + USRPT did not (all *p* > 0.05). Pre- and post-between-group differences were not significant (all *p* > 0.05; Figure 3, Table 4).

FEDH: The Time effect was significant (F_1.00_ = 36.604, *p* = 0.001, ηp^2^ = 0.404), whereas the Time × Group interaction was not (F_5.00_ = 1.407, *p* = 0.236, ηp^2^ = 0.115). Bonferroni tests indicated pre–post improvements in USRPT (*p* = 0.043, *dz* = 0.707), PLA + USRPT (*p* = 0.031, *dz* = 0.755), PRO + USRPT (*p* = 0.008, *dz* = 0.582), OMEGA + USRPT (*p* = 0.031, *dz* = 0.795), and PRO + OMEGA + USRPT (*p* = 0.001, *dz* = 2.208); CON showed no change (*p* > 0.05). Pre- and post-between-group differences were non-significant (all *p* > 0.05; Figure 3, Table 4).

### 4.2. Isokinetic and Isometric Strength Tests

**Figure 4:**
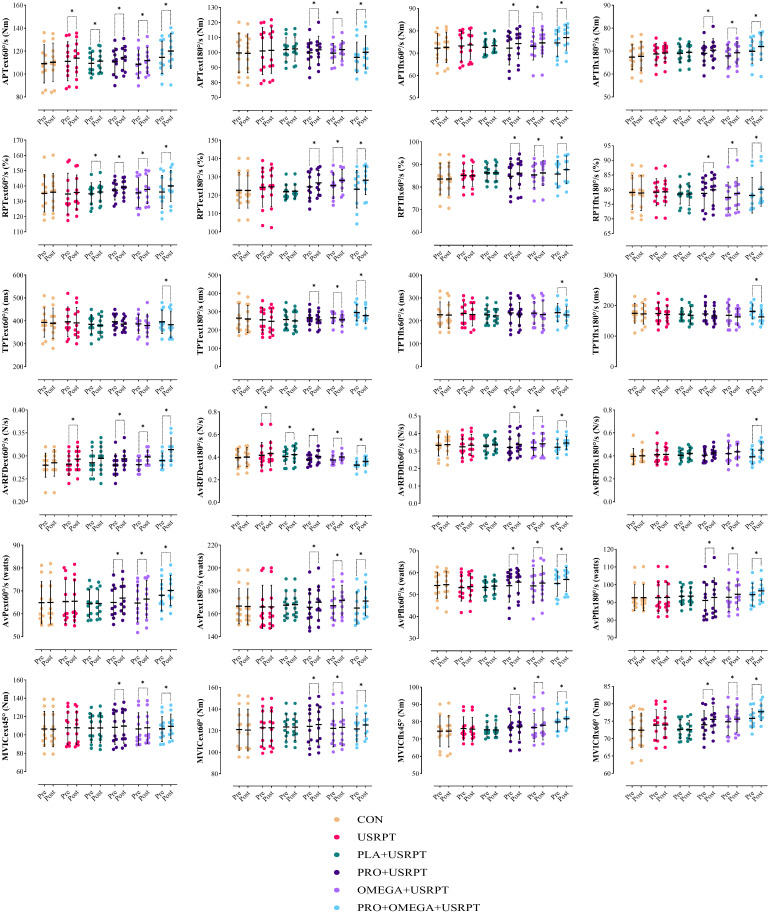
Means and standard deviations of the isokinetic and isometric strength tests results in the six groups: (control (CON), training (USRPT), placebo and training (PLA + USRPT), probiotics and training (PRO + USRPT), omega-3 and training (OMEGA + USRPT), and probiotics with omega-3 and training (PRO + OMEGA + USRPT)). APT: absolute peak torque; RPT: relative peak torque; TPT: time-to-peak torque; AvRFD: average rate of force development; AvP: average power; MVIC: maximum voluntary isometric contraction; ext: extension; flx: flexion. *: Significant difference compared with the pre-test.

APText60°/s. The two-way repeated-measures model yielded a significant Time effect (F_1.00_ = 100.238, *p* = 0.001, ηp^2^ = 0.650) and a significant Time × Group interaction (F_5.00_ = 4.019, *p* = 0.004, ηp^2^ = 0.271). Post hoc (Bonferroni) comparisons showed pre→post increases in USRPT (*p* = 0.001, *dz* = 0.860), PLA + USRPT (*p* = 0.013, *dz* = 1.040), PRO + USRPT (*p* = 0.001, *dz* = 1.715), OMEGA + USRPT (*p* = 0.001, *dz* = 1.739), and PRO + OMEGA + USRPT (*p* = 0.001, *dz* = 2.037), with CON unchanged (*p* > 0.05). Pe- and post-between-group differences were not significant (all *p* > 0.05; Figure 4, Table 4).

APText180°/s: There were significant Time (F_1.00_ = 50.738, *p* = 0.001, ηp^2^ = 0.484) and Time × Group (F_5.00_ = 9.075, *p* = 0.001, ηp^2^ = 0.457) effects. Improvements from pre- to post-intervention were detected in PRO + USRPT (*p* = 0.001, *dz* = 1.279), OMEGA + USRPT (*p* = 0.001, *dz* = 1.516), and PRO + OMEGA + USRPT (*p* = 0.001, *dz* = 1.932); CON, USRPT, and PLA + USRPT did not change (all *p* > 0.05). Between-group contrasts at each time point were non-significant (all *p* > 0.05; Figure 4, Table 4).

APTflx60°/s: A significant Time effect (F_1.00_ = 62.892, *p* = 0.001, ηp^2^ = 0.538) and Time × Group interaction (F_5.00_ = 4.566, *p* = 0.002, ηp^2^ = 0.297) was observed. Increases occurred in PRO + USRPT (*p* = 0.001, *dz* = 1.587), OMEGA + USRPT (*p* = 0.001, *dz* = 1.443), and PRO + OMEGA + USRPT (*p* = 0.001, *dz* = 1.717); CON, USRPT, and PLA + USRPT showed no change (all *p* > 0.05). Pre- and post-between-group differences were not significant (all *p* > 0.05; Figure 4, Table 4).

APTflx180°/s: The Time effect (F_1.00_ = 33.931, *p* = 0.001, ηp^2^ = 0.386) and Time × Group interaction (F_5.00_ = 2.646, *p* = 0.033, ηp^2^ = 0.197) were significant. Post hoc tests indicated pre→post gains in PRO + USRPT (*p* = 0.002, *dz* = 0.854), OMEGA + USRPT (*p* = 0.002, *dz* = 1.295), and PRO + OMEGA + USRPT (*p* = 0.001, *dz* = 1.733); CON, USRPT, and PLA + USRPT remained unchanged (all *p* > 0.05). Between-group comparisons were non-significant (all *p* > 0.05; Figure 4, Table 4).

RPText60°/s: Significant Time (F_1.00_ = 73.966, *p* = 0.001, ηp^2^ = 0.578) and Time × Group (F_5.00_ = 6.992, *p* = 0.001, ηp^2^ = 0.318) effects were present. RPText60°/s increased in PLA + USRPT (*p* = 0.026, *dz* = 0.606), PRO + USRPT (*p* = 0.001, *dz* = 1.482), OMEGA + USRPT (*p* = 0.001, *dz* = 1.536), and PRO + OMEGA + USRPT (*p* = 0.001, *dz* = 2.627); CON and USRPT showed no change (all *p* > 0.05). Pre- and post-between-group effects were not significant (all *p* > 0.05; Figure 4, Table 4).

RPText180°/s: Both Time (F_1.00_ = 59.852, *p* = 0.001, ηp^2^ = 0.526) and Time × Group (F_5.00_ = 12.840, *p* = 0.001, ηp^2^ = 0.543) effects were significant. Post hoc results showed improvements in PRO + USRPT (*p* = 0.001, *dz* = 1.552), OMEGA + USRPT (*p* = 0.001, *dz* = 1.569), and PRO + OMEGA + USRPT (*p* = 0.001, *dz* = 1.541), with CON, USRPT, and PLA + USRPT unchanged (all *p* > 0.05). Pre- and post-between-group differences were non-significant (all *p* > 0.05; Figure 4, Table 4).

RPTflx60°/s: There was a significant Time effect (F_1.00_ = 35.419, *p* = 0.001, ηp^2^ = 0.396) and Time × Group interaction (F_5.00_ = 10.111, *p* = 0.001, ηp^2^ = 0.484). Increases were observed in PRO + USRPT (*p* = 0.001, *dz* = 1.548), OMEGA + USRPT (*p* = 0.003, *dz* = 0.711), and PRO + OMEGA + USRPT (*p* = 0.001, *dz* = 1.829). CON, USRPT, and PLA + USRPT did not change (all *p* > 0.05). Between-group contrasts were not significant (all *p* > 0.05; Figure 4, Table 4).

RPTflx180°/s: The Time effect (F_1.00_ = 99.326, *p* = 0.001, ηp^2^ = 0.648) and Time × Group interaction (F_5.00_ = 22.364, *p* = 0.001, ηp^2^ = 0.674) were significant. Post hoc analyses showed increases in PRO + USRPT (*p* = 0.001, *dz* = 1.668), OMEGA + USRPT (*p* = 0.001, *dz* = 2.097), and PRO + OMEGA + USRPT (*p* = 0.001, *dz* = 3.866); CON, USRPT, and PLA + USRPT exhibited no change (all *p* > 0.05). Between-group differences were non-significant (all *p* > 0.05; Figure 4, Table 4).

TPText60°/s: A significant Time effect was detected (F_1.00_ = 8.791, *p* = 0.004, ηp^2^ = 0.140), while the Time × Group interaction was not significant (F_5.00_ = 0.393, *p* = 0.852, ηp^2^ = 0.035). PRO + OMEGA + USRPT showed a decrease from pre- to post-intervention (*p* = 0.032, *dz* = 0.688); CON, USRPT, PLA + USRPT, PRO + USRPT, and OMEGA + USRPT did not change (all *p* > 0.05). Pre- and post-between-group differences were not significant (all *p* > 0.05; Figure 4, Table 4).

TPText180°/s: The Time effect was significant (F_1.00_ = 31.798, *p* = 0.001, ηp^2^ = 0.371), but the Time × Group interaction was not (F_5.00_ = 0.907, *p* = 0.484, ηp^2^ = 0.077). Reductions were found in PRO + USRPT (*p* = 0.005, *dz* = 0.971), OMEGA + USRPT (*p* = 0.018, *dz* = 0.636), and PRO + OMEGA + USRPT (*p* = 0.001, *dz* = 1.466); CON, USRPT, and PLA + USRPT were unchanged (all *p* > 0.05). Between-group effects were non-significant (all *p* > 0.05; Figure 4, Table 4).

TPTflx60°/s: There was a significant Time effect (F_1.00_ = 9.464, *p* = 0.003, ηp^2^ = 0.149) and a non-significant interaction (F_5.00_ = 0.379, *p* = 0.861, ηp^2^ = 0.034). PRO + OMEGA + USRPT showed a pre→post decrease (*p* = 0.036, *dz* = 0.670); other groups (CON, USRPT, PLA + USRPT, PRO + USRPT, OMEGA + USRPT) did not change (all *p* > 0.05). Pre- and post-between-group comparisons were not significant (all *p* > 0.05; Figure 4, Table 4).

TPTflx180°/s: The Time effect was significant (F_1.00_ = 9.898, *p* = 0.003, ηp^2^ = 0.155); the Time × Group interaction was not (F_5.00_ = 1.355, *p* = 0.256, ηp^2^ = 0.111). A pre→post reduction was evident in PRO + OMEGA + USRPT (*p* = 0.001, *dz* = 2.281), with CON, USRPT, PLA + USRPT, PRO + USRPT, and OMEGA + USRPT unchanged (all *p* > 0.05). Between-group differences were non-significant (all *p* > 0.05; Figure 4, Table 4).

AvRFDext60°/s: A significant Time effect (F_1.00_ = 39.287, *p* = 0.001, ηp^2^ = 0.421) and a non-significant interaction (F_5.00_ = 1.534, *p* = 0.195, ηp^2^ = 0.124) were found. Increases occurred in USRPT (*p* = 0.042, *dz* = 0.661), PRO + USRPT (*p* = 0.010, *dz* = 0.979), OMEGA + USRPT (*p* = 0.002, *dz* = 1.084), and PRO + OMEGA + USRPT (*p* = 0.001, *dz* = 1.227); CON and PLA + USRPT did not change (all *p* > 0.05). Between-group differences were not significant (all *p* > 0.05; Figure 4, Table 4).

AvRFDext180°/s: The Time effect was significant (F_1.00_ = 45.737, *p* = 0.001, ηp^2^ = 0.459), with a non-significant interaction (F_5.00_ = 1.693, *p* = 0.152, ηp^2^ = 0.136). Pre→post increases were seen in USRPT (*p* = 0.043, *dz* = 0.795), PLA + USRPT (*p* = 0.017, *dz* = 0.832), PRO + USRPT (*p* = 0.001, *dz* = 1.133), OMEGA + USRPT (*p* = 0.001, *dz* = 1.101), and PRO + OMEGA + USRPT (*p* = 0.001, *dz* = 1.565); CON was unchanged (*p* > 0.05). Between-group effects at the pre/post time points were non-significant (all *p* > 0.05; Figure 4, Table 4).

AvRFDflx60°/s: We observed a significant Time effect (F_1.00_ = 21.954, *p* = 0.001, ηp^2^ = 0.289) without a significant interaction (F_5.00_ = 1.266, *p* = 0.292, ηp^2^ = 0.105). Increases occurred in PRO + USRPT (*p* = 0.020, *dz* = 1.367), OMEGA + USRPT (*p* = 0.005, *dz* = 1.076), and PRO + OMEGA + USRPT (*p* = 0.002, *dz* = 0.868); CON, USRPT, and PLA + USRPT did not change (all *p* > 0.05). Between-group differences were not significant (all *p* > 0.05; Figure 4, Table 4).

AvRFDflx180°/s: The Time effect was significant (F_1.00_ = 12.138, *p* = 0.001, ηp^2^ = 0.184), with no significant interaction (F_5.00_ = 2.004, *p* = 0.093, ηp^2^ = 0.156). A pre→post increase was found in PRO + OMEGA + USRPT (*p* = 0.001, *dz* = 2.518); other groups (CON, USRPT, PLA + USRPT, PRO + USRPT, OMEGA + USRPT) were unchanged (all *p* > 0.05). Between-group comparisons at each time point were non-significant (all *p* > 0.05; Figure 4, Table 4).

AvPext60°/s: Significant Time (F_1.00_ = 116.984, *p* = 0.001, ηp^2^ = 0.684) and Time × Group (F_5.00_ = 19.608, *p* = 0.001, ηp^2^ = 0.645) effects were observed. Post hoc tests indicated increases in PRO + USRPT (*p* = 0.001, *dz* = 3.277), OMEGA + USRPT (*p* = 0.001, *dz* = 2.496), and PRO + OMEGA + USRPT (*p* = 0.001, *dz* = 2.914), with CON, USRPT, and PLA + USRPT unchanged (all *p* > 0.05). Pre- and post-between-group differences were not significant (all *p* > 0.05; Figure 4, Table 4).

AvPext180°/s: There were significant Time (F_1.00_ = 160.110, *p* = 0.001, ηp^2^ = 0.748) and Time × Group (F_5.00_ = 32.626, *p* = 0.001, ηp^2^ = 0.751) effects. Pre→post gains occurred in PRO + USRPT (*p* = 0.001, *dz* = 2.499), OMEGA + USRPT (*p* = 0.001, *dz* = 4.256), and PRO + OMEGA + USRPT (*p* = 0.001, *dz* = 2.081). CON, USRPT, and PLA + USRPT did not change (all *p* > 0.05). Between-group comparisons at each time point were non-significant (all *p* > 0.05; Figure 4, Table 4).

AvPflx60°/s: Both Time (F_1.00_ = 58.502, *p* = 0.001, ηp^2^ = 0.520) and Time × Group (F_5.00_ = 4.047, *p* = 0.003, ηp^2^ = 0.273) effects were significant. Post hoc tests showed increases in PRO + USRPT (*p* = 0.001, *dz* = 0.926), OMEGA + USRPT (*p* = 0.001, *dz* = 1.627), and PRO + OMEGA + USRPT (*p* = 0.001, *dz* = 2.466); CON, USRPT, and PLA + USRPT were unchanged (all *p* > 0.05). Between-group differences were not significant (all *p* > 0.05; Figure 4, Table 4).

AvPflx180°/s: The Time effect (F_1.00_ = 74.778, *p* = 0.001, ηp^2^ = 0.581) and Time × Group interaction (F_5.00_ = 12.563, *p* = 0.001, ηp^2^ = 0.538) were significant. Pre→post increases were observed in PRO + USRPT (*p* = 0.001, *dz* = 1.257), OMEGA + USRPT (*p* = 0.001, *dz* = 1.330), and PRO + OMEGA + USRPT (*p* = 0.001, *dz* = 3.517). CON, USRPT, and PLA + USRPT showed no change (all *p* > 0.05). Between-group comparisons were non-significant (all *p* > 0.05; Figure 4, Table 4).

MVICext45°: There were significant Time (F_1.00_ = 85.958, *p* = 0.001, ηp^2^ = 0.614) and Time × Group (F_5.00_ = 25.552, *p* = 0.001, ηp^2^ = 0.703) effects. MVICext45° increased in PRO + USRPT (*p* = 0.001, *dz* = 1.912), OMEGA + USRPT (*p* = 0.001, *dz* = 1.912), and PRO + OMEGA + USRPT (*p* = 0.001, *dz* = 6.418); CON, USRPT, and PLA + USRPT did not change (all *p* > 0.05). Between-group differences were not significant (all *p* > 0.05; Figure 4, Table 4).

MVICext60°: The Time effect (F_1.00_ = 43.292, *p* = 0.001, ηp^2^ = 0.445) and Time × Group interaction (F_5.00_ = 20.314, *p* = 0.001, ηp^2^ = 0.653) were significant. Pre→post gains were noted in PRO + USRPT (*p* = 0.001, *dz* = 3.061), OMEGA + USRPT (*p* = 0.008, *dz* = 1.289), and PRO + OMEGA + USRPT (*p* = 0.001, *dz* = 1.722); CON, USRPT, and PLA + USRPT were unchanged (all *p* > 0.05). Between-group comparisons at the pre/post time points were non-significant (all *p* > 0.05; Figure 4, Table 4).

MVICflx45°: Significant Time (F_1.00_ = 41.585, *p* = 0.001, ηp^2^ = 0.435) and Time × Group (F_5.00_ = 16.361, *p* = 0.001, ηp^2^ = 0.602) effects were observed. MVICflx45° increased in PRO + USRPT (*p* = 0.001, *dz* = 0.960), OMEGA + USRPT (*p* = 0.001, *dz* = 2.624), and PRO + OMEGA + USRPT (*p* = 0.001, *dz* = 2.786); CON, USRPT, and PLA + USRPT did not change (all *p* > 0.05). Between-group differences were non-significant (all *p* > 0.05; Figure 4, Table 4).

MVICflx60°: The Time effect (F_1.00_ = 57.114, *p* = 0.001, ηp^2^ = 0.514) and Time × Group interaction (F_5.00_ = 17.620, *p* = 0.001, ηp^2^ = 0.620) were significant. Post hoc tests showed increases in PRO + USRPT (*p* = 0.001, *dz* = 2.527), OMEGA + USRPT (*p* = 0.001, *dz* = 1.351), and PRO + OMEGA + USRPT (*p* = 0.001, *dz* = 4.371); CON, USRPT, and PLA + USRPT were unchanged (all *p* > 0.05). Between-group differences at the pre/post time points were not significant (all *p* > 0.05; Figure 4, Table 4).

## 5. Discussion

Our findings demonstrated that combining probiotics with omega-3 fatty acids (a synbiotic approach) during intensive USRPT elicited superior improvements in upper-body muscular strength and endurance compared with either supplement alone or training without supplementation. All supplemented groups (probiotics only, omega-3 only, and especially the combined synbiotics) showed significant pre- to post-training gains in functional tests—including handgrip strength (both dominant and non-dominant), medicine-ball-throw distance, and dead-hang duration—as well as in isokinetic and isometric shoulder strength measures. In contrast, the placebo and training-only groups exhibited minimal changes. Notably, the probiotic + omega-3 group achieved the most substantial enhancements across nearly all metrics, indicating a synergistic effect beyond additive benefits. These results underscore the potential of targeting the gut–muscle axis through coordinated nutritional strategies to amplify training adaptations in elite athletes. They align with emerging evidence that gut-focused interventions can influence skeletal muscle performance, providing practical insight for sports nutrition and conditioning programs.

Gut–Muscle Axis and Mechanistic Insights: The significant benefits observed from synbiotic supplementation can be understood through the gut–muscle axis, where gut microbiota and their metabolites influence host inflammation, immunity, and nutrient metabolism, ultimately affecting muscle function [59]. High-intensity exercise is known to disrupt gut homeostasis transiently—for example, rigorous training can increase intestinal permeability and alter microbiota composition, which may impair recovery and performance. Athletes also tend to harbor distinct gut microbiomes associated with enhanced metabolic capacity [60]. In this context, probiotic supplementation has been shown to support gut-barrier integrity and reduce circulating endotoxin levels. In particular, strains such as *Lactiplantibacillus plantarum* (formerly *Lactobacillus plantarum*) and *Bifidobacterium longum* can enhance intestinal integrity and reduce circulating lipopolysaccharide (LPS) levels, thereby mitigating systemic low-grade inflammation [61,62]. A more intact gut barrier and reduced endotoxemia would lessen inflammatory interference with muscle [63]. Additionally, probiotic-induced shifts in microbiota composition and the production of short-chain fatty acids (SCFAs) may directly support muscle energy metabolism and anti-inflammatory signaling (for example, SCFAs produced by beneficial *Lactobacillus* and *Bifidobacterium* species). Beyond SCFAs, microbiota-derived metabolites, such as 3-(4-hydroxy-3-methoxyphenyl) propionic acid, have been reported to reduce oxidative stress and shift the muscle fiber composition, thereby supporting skeletal muscle function [64]. Recent reviews offer detailed mechanistic insights into how microbiota-derived metabolites influence muscle physiology. Furthermore, specific probiotic strains enhance anti-inflammatory responses, such as by increasing IL-10 levels and regulatory T-cell activity, and reduce exercise-induced increases in TNF-α and oxidative stress. These effects can help mitigate training-induced inflammation and support greater strength and endurance gains [65,66,67]. Consistent with these mechanisms, swimmers receiving synbiotic supplementation in our study achieved significantly greater improvements in upper-body functional strength and shoulder torque measures compared with those receiving probiotics alone, omega-3 fatty acids alone, or training alone. This highlights the potential of gut-targeted strategies to optimize training adaptations.

Probiotics may also enhance nutrient absorption and bioavailability, supporting muscle anabolism. In a trial by Jäger et al. (2020), a multi-strain probiotic increased amino acid uptake from a plant protein supplement, indicating that gut microbes can facilitate nutrient utilization [68]. In our study, improved amino acid availability from the diet may have led to greater muscle protein synthesis and repair, particularly when combined with intense training. This may partly explain why the probiotic-supplemented swimmers achieved higher gains in peak torque, average power, and grip strength—outcomes that depend on effective muscle remodeling and growth. Improved nutrient status via a healthier gut microbiome is particularly relevant given the caloric and protein demands of elite swimmers. Indeed, recent reviews underscore that probiotics can help maintain lean mass and strength by supporting protein metabolism and blunting catabolic signals in athletes [69,70]. Our results add to this evidence, as the probiotic-only group outperformed the placebo group in multiple strength measures, suggesting that the gut-mediated nutritional support conferred an ergogenic advantage.

Complementing the probiotic effects, omega-3 (EPA/DHA) provided synergistic benefits through anti-inflammatory and muscle-protective mechanisms, including incorporation into membranes and a shift in eicosanoids toward less inflammatory species, which reduced exercise-induced inflammation and soreness [71]. In the present trial, swimmers receiving omega-3 exhibited greater improvements in explosive power and isokinetic torque than unsupplemented athletes, likely because EPA/DHA dampened the post-exercise rise in pro-inflammatory cytokines (such as IL-6 and TNF-α) that can otherwise impair muscle contractility and delay recovery. This anti-inflammatory action is supported by previous findings that omega-3 supplementation lowers circulating inflammatory markers after intense training and can expedite strength recovery [72]. Moreover, omega-3s enhance the structural and functional properties of muscle cell membranes, improving membrane fluidity and the function of ion channels involved in excitation–contraction coupling [73]. Practically, this may lead to improved neuromuscular efficiency and faster force generation, consistent with our findings of a shorter time-to-peak torque and higher force development rate in the omega-3 group compared with training alone. Omega-3 fatty acids are also known to activate anabolic signaling pathways like AKT/mTOR and to promote muscle protein synthesis while reducing proteolysis, resulting in modest improvements in muscle strength and function, even among older adults [72]. Our results extend these benefits to young, highly-trained athletes, suggesting that even in elite muscle, omega-3s can confer an extra boost to strength adaptations. This effect was especially evident at higher movement velocities (e.g., 180°/s) and in isometric tests in our study, indicating that omega-3s may support both dynamic power output and static force maintenance. Notably, however, the omega-3 group’s gains still did not equal those of the combined synbiotic group in many measures—reinforcing that multiple complementary pathways were likely engaged when probiotics and omega-3s were co-administered.

Synergistic Synbiotic Interaction: Combining probiotics and omega-3 resulted in the most pronounced improvements across all performance outcomes, suggesting an additive or synergistic interaction between gut microbiota-targeted and anti-inflammatory interventions [63]. This synbiotic concept—traditionally referring to probiotic-plus-prebiotic combinations—can reasonably be expanded to include probiotics and omega-3 fatty acids, based on recent evidence that omega-3s can also act as microbiota-modulating agents. Omega-3 intake has been linked to increased gut microbial diversity and the promotion of health-supporting bacteria, effectively showing prebiotic-like properties in humans. Some expert consensus definitions of prebiotics have broadened to encompass certain bioactive compounds like PUFAs that positively influence the microbiome [74]. Thus, co-supplementing probiotics with omega-3 fatty acids may create a positive feedback loop: the probiotic strains improve the gut environment and enhance nutrient absorption, allowing omega-3s to be utilized optimally. In contrast, omega-3s create systemic anti-inflammatory conditions and possibly foster a gut microbiome profile that supports probiotic colonization and metabolic activity. The outcome is a complementary reduction in systemic inflammation and oxidative stress alongside improved nutrient and energy availability for muscle tissue. This could explain why the combined group in our study not only had the highest absolute gains but also exhibited unique benefits—such as faster neuromuscular response (significantly shorter time-to-peak torque)—that were not as evident with either supplement alone. The dual intervention likely enabled athletes to train at high intensity with less accumulated fatigue and muscle damage, thereby amplifying training effectiveness over the 8 weeks. Supporting this interpretation, Maymandinejad et al. (2025)—in a parallel trial on sprint swimmers—reported that only the probiotic + omega-3 co-supplementation led to significant improvements in 50 m and 100 m freestyle race times, whereas probiotics or omega-3 alone produced smaller or nonsignificant performance gains [38]. In that study, the combined synbiotic group also demonstrated better improvements in anaerobic power indices and reaction time, emphasizing that the synergy between probiotics and omega-3 benefits both muscular strength and functional performance outcomes [38]. Mechanistically, our data and others suggest that probiotics prime the gut–muscle axis by enhancing amino acid uptake and immune homeostasis, while omega-3s directly support muscle cell adaptation and quell inflammation; together, these interventions likely target distinct but intersecting pathways to maximize muscle function. The concept of leveraging multiple nutritional ergogenic aids concurrently is further bolstered by the safety and complementary modes of action of probiotics and omega-3s, in contrast to single-supplement approaches that may address only one aspect of the physiological stress from training.

Comparison with Recent Literature: Our results align with a growing body of literature (2020–2025) that investigates multi-ingredient strategies to enhance athletic performance. In particular, several studies by Hemmatinafar and colleagues corroborate the benefits of combining probiotics with other nutrients in trained populations. For example, Imanian et al. (2024) reported that in male soccer players, four weeks of probiotics plus casein protein supplementation resulted in significantly longer time-to-exhaustion and greater improvements in ventilatory thresholds compared with a placebo, whereas probiotics or casein protein alone had more modest effects [18]. This suggests a potentiation of endurance capacity when gut health support is paired with protein nutrition, analogous to the synergistic effects on endurance and strength gains observed with probiotics and omega-3s. Similarly, Sadeghi et al. (2025) observed that adding a probiotic mix to a pre-sleep casein routine significantly enhanced anaerobic power and lower-body muscle strength in soccer players compared with casein alone [44]. They suggested this improvement results from better muscle recovery overnight, emphasizing that probiotic-driven gut and immune system modulation can boost the muscle-building effects of protein. In another recent recovery-focused study, Ahmadi et al. (2024) found that female futsal athletes who took combined omega-3 and whey protein supplements for eight weeks showed greater increases in muscle strength and less delayed-onset muscle soreness after intense exercise than those taking only one of the supplements [73]. The co-supplemented group’s superior recovery was linked to reduced inflammation and muscle damage, mirroring the theme that anti-inflammatory nutrients (like fish oil) and anabolic substrates (protein, or in our case, the amino acids made more available by probiotics) work best in tandem. Together with the present trial, these studies underscore that targeting multiple biological pathways yields more pronounced performance benefits. They collectively support the paradigm that synbiotic or combination supplementation (probiotics with proteins, or probiotics with omega-3 fatty acids) can improve various facets of athletic performance—from endurance and sprint capacity to strength and recovery.

Beyond our studies, other contemporary research aligns with this notion. A 2022 systematic review by Prokopidis et al. concluded that probiotics have a positive, if moderate, effect on muscle mass and strength maintenance, particularly in older adults [75]. An updated review of probiotic use in sports science similarly suggests that probiotics can mitigate some of the negative impacts of strenuous exercise (such as excessive inflammation or immune disturbance), thereby indirectly enhancing performance [70]. Moreover, several trials have documented that probiotic supplementation can boost exercise performance and support immune function in athletes without adverse effects, while omega-3 fatty acids on their own can reduce exercise-induced muscle damage and inflammation. Our study adds novel evidence that when these two interventions are combined, the benefits are amplified in an elite sports setting. This contributes to the evolving field of exercise–microbiome research by illustrating a clear example of how modulating the gut microbiota and systemic inflammation together can translate into measurable improvements in human performance.

The multi-strain probiotic formulation used in this study was carefully chosen based on its potential to work synergistically in improving gastrointestinal health, supporting immune function, and enhancing muscular strength. Although there is limited direct evidence on this specific probiotic combination in athletic groups, previous research has highlighted the individual benefits of its component strains. For example, *Lactiplantibacillus plantarum* BP06 has shown significant anti-inflammatory properties and the ability to strengthen intestinal barrier integrity, which can improve nutrient absorption and aid recovery from exercise-related physiological stress [76]. Likewise, *Bifidobacterium longum* BL03 has been implicated in regulating immune responses and attenuating exercise-associated gastrointestinal disturbances—factors essential for preserving muscular function during prolonged physical activity [77,78]. Additionally, strains such as *Lactobacillus acidophilus* BA05 and *Bifidobacterium breve* BB02 have been associated with reductions in oxidative stress and improvements in immune competency, both of which contribute to the mitigation of fatigue and the enhancement of post-exercise recovery in physically active populations [79,80]. Furthermore, *Lactobacillus bulgaricus* BD08 and *Streptococcus thermophilus* BT01, commonly incorporated into multi-strain probiotic preparations, are known to stabilize gut microbiota composition and facilitate protein metabolism, thereby supporting the adaptive responses required in high-intensity training protocols [81]. These mechanistic insights clarify the improvements in muscular strength, especially in isokinetic performance, observed in our study. Although the actions of its components support the effectiveness of our probiotic blend, further research is needed to explore its long-term benefits and to determine the optimal application in sport-specific situations. Future studies should focus on understanding how these strains interact, thereby enhancing our knowledge of their combined effects on endurance, recovery, and overall athletic performance.

In the functional strength assessments, both dominant (DHGS) and non-dominant (NDHGS) handgrip strengths significantly improved in the PRO + USRPT, OMEGA + USRPT, and PRO + OMEGA + USRPT groups. These results align with previous studies suggesting that probiotics can enhance muscle strength by influencing the gut–muscle axis, increasing amino acid absorption, and decreasing systemic inflammation [75]. However, a population study noted that plasma omega-3 levels did not correlate with handgrip strength in people over 50 [82], indicating that the strength-enhancing effects of omega-3 may depend on factors such as age, initial nutritional status, or training stimuli. The throwing-medicine-ball test (upper-body power) and dead-hang tests (muscular endurance in straight- and flexed-arm positions) also improved significantly from pre- to post-intervention in all supplement groups combined with USRPT, but not in the placebo or USRPT-only groups. This is in line with reports that probiotic supplementation may attenuate the adverse effects of prolonged high-intensity exercise and thereby improve aspects of sports performance [61]. In contrast, Townsend et al. observed that a 12-week probiotic regimen did not significantly affect body composition, performance, hormonal status, or gut permeability in Division I athletes; however, it did attenuate circulating TNF-α levels during training [83]. Such discrepancies in the literature highlight that probiotic effects can vary with the strains used, the athlete population, and the measured outcomes—reinforcing the importance of multi-strain approaches and combined interventions, as used in our study.

Probiotics have been shown to influence various physiological processes that contribute to muscle strength and recovery. Supplementation with probiotics, particularly multi-strain blends, can improve gut health, modulate immune function, and reduce systemic inflammation—all of which play significant roles in muscle recovery and performance [69,70]. Specific strains such as *Lactiplantibacillus plantarum* and *Bifidobacterium longum* (like those included in our formula) have demonstrated benefits in reducing exercise-induced inflammation and enhancing the body’s ability to recover from high-intensity exercise [84]. Additionally, the improvements in gut microbiota composition facilitated by probiotics may support better nutrient absorption (e.g., of amino acids and minerals), thus contributing to more effective muscle repair and growth [79]. In the present study, the inclusion of probiotics alongside USRPT likely supported these processes, resulting in greater gains in functional performance—particularly evident in the handgrip strength tests.

Omega-3 fatty acids, particularly EPA and DHA, are recognized for their anti-inflammatory effects and support in muscle recovery [76]. Supplementing with omega-3 has been demonstrated to boost muscle protein synthesis, lessen muscle damage caused by exercise, and enhance strength results after intense workouts [72]. In the context of USRPT, omega-3s may help to mitigate muscle soreness and accelerate recovery between sessions, contributing to sustained improvements in strength over time [85]. Omega-3s are also known to influence cell membrane integrity and reduce oxidative stress, which can be particularly beneficial for muscle function and recovery during high-intensity training [85]. The observed benefits in our swimmers’ functional strength indicators likely reflect these cumulative effects of omega-3 supplementation, supporting the notion that these fatty acids can bolster performance in strength-dependent tasks.

On the other hand, when probiotics and omega-3 were administered together, the combined effects on muscle strength were greater than when each supplement was used separately. This synergy could be attributed to the complementary roles of each substance in reducing inflammation and enhancing nutrient absorption and muscle repair [74]. For example, while omega-3s reduce inflammation and muscle damage, probiotics improve gut health and immune homeostasis, further supporting muscle recovery and function [71,85]. This combination may have provided a more robust foundation for strength gains in both the dominant and non-dominant handgrip tests, as both supplements act to reduce systemic stressors that can impair muscle performance. In contrast, the control group and the USRPT-only group exhibited much less pronounced improvements in these functional strength indicators, which can be attributed to the absence of any supplementation aimed at blunting inflammation or improving recovery. This suggests that probiotics and omega-3 fatty acids—either separately or, especially, together—are particularly beneficial in enhancing muscle strength when combined with high-intensity training such as USRPT [2]. The interaction of targeted nutritional support with rigorous training likely potentiated the positive adaptations in strength, underscoring the importance of an integrated approach to maximize the benefits of intense training regimens.

In the isokinetic tests, both the absolute (APT) and relative peak torque (RPT) of the shoulder extensors and flexors at 60°/s and 180°/s improved significantly in the PRO + USRPT, OMEGA + USRPT, and, especially, the PRO + OMEGA + USRPT groups. The combined group achieved the most significant gains, likely due to enhanced Type II fiber recruitment and reduced exercise-induced oxidative stress. Time-to-peak torque (TPT) decreased only in the combined group (~13 ms faster), indicating better motor unit synchronization and neural drive. Correspondingly, the average rate of force development (AvRFD) and average power (AvP) improved in all supplemented groups, most notably in the PRO + OMEGA + USRPT group, with larger gains at 180°/s reflecting enhanced high-velocity force generation. These findings suggest that omega-3 supports muscle energetics while probiotics reduce systemic inflammation, together improving nutrient utilization and recovery. Isometric strength (MVIC) at 45° and 60° angles increased significantly only in the PRO + OMEGA + USRPT group, whereas other groups showed minimal or no changes. This implies that the combined anti-inflammatory, anabolic, and neuromuscular benefits of probiotics plus omega-3 were necessary for substantial static strength gains. Little to no improvements occurred in the PLA + USRPT, USRPT-only, or control groups, highlighting that the observed performance gains stemmed primarily from the supplementation interventions rather than training alone. Probiotic supplementation may enhance performance by reducing systemic inflammation, strengthening the intestinal barrier, and improving amino acid absorption—particularly of the BCAAs essential for muscle protein synthesis [68]. These effects likely contributed to the observed increases in peak torque and power in the PRO + USRPT and PRO + OMEGA + USRPT groups. Probiotics also mitigate exercise-induced oxidative stress and enhance mitochondrial function, thereby further supporting muscle force and resistance to fatigue [86]. Omega-3 fatty acids (EPA and DHA) enhance these effects by stabilizing cell membranes, lowering pro-inflammatory cytokines like TNF-α and IL-6, and stimulating anabolic signaling pathways such as mTORC1. This leads to improved neuromuscular function and strength outcomes, including MVIC and sustained power [71,73].

It should be noted that the USRPT regimen itself—a high-intensity interval training model based on repeated race-pace efforts—is effective in stimulating the recruitment of fast-twitch muscle fibers, increasing neuromuscular coordination, and enhancing anaerobic capacity [26]. Repeated high-intensity efforts are known to improve the rate of force development (as reflected by the AvRFD) and promote adaptations in motor-neuron firing rates, which likely underpin the gains observed in both the concentric peak torque and MVIC measures [87]. In our trial, the addition of probiotic and omega-3 supplementation appears to have augmented these training-induced adaptations via multiple pathways: by improving recovery (through faster resolution of inflammation and muscle damage), reducing exercise-induced oxidative stress, and enhancing anabolic signaling for muscle repair. This multi-modal intervention supported faster strength development and better recovery, as evident in the significantly lower TPT and higher post-training APT and RPT across the supplemented groups compared with the CON and USRPT-only groups. Additionally, the combined use of probiotics and omega-3s likely had a synergistic effect on muscle strength. Probiotics enhance nutrient absorption, while omega-3s improve muscle cells’ responsiveness to nutrients (including amino acids and insulin), amplifying mTORC1 signaling and protein synthesis. Both interventions also reduce systemic inflammation and oxidative damage, leading to improved muscle repair and functional capacity. These combined effects underpin the superior performance observed in the PRO + OMEGA + USRPT group—particularly for MVIC, APT, and AvP outcomes. The coordinated modulation of immune function, oxidative stress, and metabolic signaling pathways by this dual-supplement strategy represents a promising approach for maximizing training adaptations, and it warrants further investigation in sports performance research.

Given the modest sample size (*n* = 10/group), we interpreted the findings using both relative changes and effect sizes to distinguish between statistical and practical relevance. The synbiotic + USRPT group showed the most considerable improvements in sprint-relevant, high-velocity shoulder outcomes: AvRFDflx180°/s increased by +15.38% (others ≤ 5%), APText180°/s rose by +3.53% (others ≤ 2.44%), and AvPext180°/s increased by +3.70% (others ≤ 2.81%), while the time-to-peak torque decreased most strongly (TPText180°/s −5.74%, TPTflx180°/s −9.94%), collectively indicating faster force rise and greater dynamic power, consistent with the sprint mechanics. These patterns align with the updated Results reporting ηp^2^ for ANOVA effects and within-group Cohen’s d (*dz*), where large within-group effects are observed for several synbiotic arms (e.g., DHGS *dz* ≈ 1.25; FEDH *dz* ≈ 2.21), while interaction effects for field tests are minor or nonsignificant (e.g., TMB: Time × Group ηp^2^ = 0.029, ns), underscoring limited between-group differentiation for non-stroke-embedded tasks. In practical terms, although handgrip (+8.80%) and medicine-ball throw (+2.37%) improved numerically in the synbiotic + USRPT group, their ecological linkage to propulsion is weaker (isometric/distal for handgrip; global and non-stroke-specific for TMB), and the minor interaction effects suggest limited applied importance for elite sprint performance. By contrast, the larger, directionally consistent gains in high-velocity torque, power, rate of force development, and reduced time-to-peak torque are directly aligned with the explosive demands of starts, turns, and early-cycle acceleration in freestyle sprinting and therefore represent the most meaningful adaptations observed.

## 6. Limitations and Future Directions

Limitations: This trial should be interpreted in light of several constraints. First, the sample size was modest (*n* = 10 per group), which limited precision and the detection of small effects. Second, the cohort comprised only male elite sprint freestyle (front crawl) swimmers; the absence of women and recreational/other-stroke athletes restricts the generalizability and precludes sex- or stroke-based analyses. Third, we did not stratify by age or test age × intervention interactions, so potential developmental differences could not be resolved. Fourth, the intervention period was 8 weeks, capturing short-term adaptations without follow-up on durability or dose–response. Fifth, although we monitored diet using an FFQ (at baseline and week 8) and weekly checklists and asked athletes to maintain their habitual intake and to avoid initiating probiotic/omega-3 supplements, the background diet was not biomarker-verified (e.g., omega-3 index, fecal measures), so residual dietary confounding cannot be excluded. Sixth, mechanistic inference is limited: we did not perform direct microbiome or metabolite assays (e.g., 16S/shotgun sequencing, SCFAs, bile acids, indoles, TMAO) or assay for barrier/inflammation markers (e.g., zonulin, LPS/LBP, IL-6, TNF-α, hsCRP), so any reference to gut–muscle-axis modulation is hypothesis-generating rather than causal. Seventh, the testing battery relied primarily on dry-land proxies of shoulder strength/power; we omitted in-water, stroke-embedded assessments (e.g., sEMG synchronized with kinematics, tethered/power-rack, start/turn force–time), which would offer greater ecological validity. Finally, some field outcomes that are not stroke-embedded (e.g., handgrip, medicine-ball throw) showed small effect sizes and/or small relative changes, suggesting limited practical relevance for elite sprint performance compared with the larger, directionally consistent gains observed in the high-velocity isokinetic torque/power, rate of force development, and time-to-peak torque.

Future directions. Subsequent work should (i) include female athletes with cycle-phase tracking and be powered for sex-specific effects; (ii) broaden the sampling frame to recreational swimmers and additional strokes and stratify by age to test age × intervention/test interactions; (iii) extend the intervention duration with post-intervention follow-up to examine the durability of adaptations and evaluate continuous vs. cyclic supplementation and dose/type timing (EPA/DHA composition, probiotic strain/dose) relative to USRPT microcycles; (iv) implement tighter dietary control and verification, including repeated 24 h recalls/food logs plus biomarkers (omega-3 index; fecal SCFAs) to minimize confounding and to quantify exposure; (v) integrate multi-omics (stool 16S/shotgun sequencing, targeted/untargeted metabolomics) together with barrier and inflammatory markers and apply mediation analyses to test whether microbe/metabolite changes statistically account for strength adaptations; and (vi) add in-water, stroke-embedded performance tests—notably sEMG in water synchronized with kinematics, tethered or power-rack force testing, and start/turn force–time profiling—to link laboratory strength gains to technical efficiency and race-determinant mechanics. Collectively, these refinements will strengthen the external validity, clarify mechanisms, and determine the extent to which laboratory-based improvements translate into meaningful, stroke-specific performance benefits.

## 7. Conclusions

This study demonstrates that synbiotic supplementation with probiotics and omega-3 fatty acids, when combined with ultra-short race-pace training (USRPT), produces superior improvements in upper-body strength, power, and endurance compared with training alone or with single interventions. The synergistic benefits likely arise from complementary mechanisms; probiotics enhance gut-barrier integrity, nutrient absorption, and immune regulation, while omega-3 fatty acids attenuate inflammation and support muscle anabolic processes. Together, these effects amplified training adaptations, as evidenced by significant interaction effects on multiple functional, isokinetic, and isometric performance outcomes in elite sprint swimmers. These findings highlight the potential of targeting the gut–muscle axis as part of a comprehensive strategy to optimize responses to high-intensity training. Incorporating synbiotic supplementation into personalized training programs may provide athletes with a practical means of enhancing explosive performance and muscular endurance while supporting recovery and overall physiological resilience. Future research should explore underlying microbiome-mediated mechanisms and assess the applicability of this combined approach across diverse athletic populations and performance settings.

## Figures and Tables

**Table 1:** The anthropometric data of participants.

Characteristic	Mean ± SD (*n* = 60)
Age (years)	19.20 ± 3.64
Height (cm)	182.20 ± 5.21
Weight (kg)	81.60 ± 4.42

**Table 2:** Strains and dosage per one capsule (200 mg) of the probiotics used in the present study.

Strains	Dosage (CFU)
*Lactiplantibacillus plantarum* BP06	0.43 × 10^11^
*Lacticaseibacillus casei* BP07	0.65 × 10^11^
*Lactobacillus acidophilus* BA05	0.94 × 10^11^
*Lactobacillus bulgaricus* BD08	0.36 × 10^11^
*Bifidobacterium infantis* BI04	0.57 × 10^11^
*Bifidobacterium longum* BL03	0.73 × 10^11^
*Bifidobacterium breve* BB02	0.62 × 10^11^
*Streptococcus thermophilus* BT01	0.20 × 10^11^
Total	4.5 × 10^11^

**Table 3:** Means, % relative changes, and standard deviation (SD) of the pre-test and post-test measured variables.

			CON	USRPT	PLA + USRPT	OMEGA + USRPT	PRO + USRPT	PRO + OMEGA + USRPT
DHGS (kg)	Mean	Pre	52.60	52.50	53.40	52.10	51.80	52.30
	SD		4.37	4.08	5.77	4.48	5.32	6.05
	Mean	Post	53.50	54.10	55.00	55.90	54.70	56.90
	SD		4.50	3.51	6.09	6.13	5.81	6.69
	%Δ (post vs. pre)		1.71%	3.05%	3.00%	7.29%	5.60%	8.80%
NDHGS (kg)	Mean	Pre	50.10	50.60	50.10	50.80	50.50	50.40
	SD		4.33	4.62	5.48	4.93	4.74	5.18
	Mean	Post	51.10	51.20	49.90	53.10	50.80	52.40
	SD		5.21	4.36	6.96	5.36	5.32	5.23
	%Δ (post vs. pre)		2.00%	1.19%	−0.40%	4.53%	0.59%	3.97%
TMB (m)	Mean	Pre	9.85	9.74	9.73	9.79	9.84	9.71
	SD		1.08	0.76	0.81	0.89	0.82	0.78
	Mean	Post	10.00	9.87	9.83	10.01	10.02	9.94
	SD		0.96	0.89	0.76	0.83	0.76	0.77
	%Δ (post vs. pre)		1.52%	1.33%	1.03%	2.25%	1.83%	2.37%
SEDH (kg)	Mean	Pre	98.30	95.50	97.90	96.40	93.10	96.60
	SD		26.17	24.61	25.97	30.46	24.66	26.03
	Mean	Post	98.70	97.00	99.40	98.20	94.90	98.90
	SD		25.57	23.68	24.81	28.84	23.71	25.97
	%Δ (post vs. pre)		0.41%	1.57%	1.53%	1.87%	1.93%	2.38%
FEDH (kg)	Mean	Pre	50.10	48.40	49.80	49.80	47.40	48.20
	SD		13.41	10.36	10.52	13.13	12.44	11.93
	Mean	Post	50.80	49.90	51.40	51.80	49.00	51.50
	SD		12.34	10.20	10.27	13.03	12.58	11.83
	%Δ (post vs. pre)		1.40%	3.10%	3.21%	4.02%	3.38%	6.85%
APText60°/s (Nm)	Mean	Pre	109.03	111.10	109.32	110.68	108.55	114.54
	SD		16.79	17.0	9.31	12.20	12.23	14.11
	Mean	Post	110.26	113.87	111.22	114.17	111.74	120.04
	SD		16.76	15.80	9.66	10.71	11.39	15.35
	%Δ (post vs. pre)		1.13%	2.49%	1.74%	3.15%	2.94%	4.80%
APText180°/s (Nm)	Mean	Pre	99.70	101.06	101.92	99.84	99.49	96.73
	SD		13.21	15.07	7.88	9.08	6.61	10.18
	Mean	Post	99.45	101.43	102.29	101.81	101.92	100.14
	SD		12.90	15.37	8.03	8.99	6.24	11.05
	%Δ (post vs. pre)		−0.25%	0.37%	0.36%	1.97%	2.44%	3.53%
APTflx60°/s (Nm)	Mean	Pre	72.22	73.21	72.56	72.27	73.12	74.57
	SD		6.60	6.76	3.67	7.53	6.14	5.97
	Mean	Post	72.60	73.73	73.35	74.28	74.64	77.00
	SD		6.77	6.47	3.17	6.75	6.36	5.67
	%Δ (post vs. pre)		0.53%	0.71%	1.09%	2.78%	2.08%	3.26%
APTflx180°/s (Nm)	Mean	Pre	67.36	68.76	69.03	69.01	67.88	69.93
	SD		5.40	4.60	4.02	5.16	4.91	5.39
	Mean	Post	67.75	69.18	69.49	70.40	69.26	71.99
	SD		6.31	3.80	3.92	5.00	4.87	5.99
	%Δ (post vs. pre)		0.58%	0.61%	0.67%	2.01%	2.03%	2.95%
RPText60°/s (%)	Mean	Pre	135.16	134.77	134.87	137.25	135.60	136.05
	SD		12.23	13.53	7.28	6.39	9.79	10.92
	Mean	Post	135.93	135.76	136.08	139.20	137.81	140.03
	SD		12.31	11.44	6.91	5.31	9.31	10.16
	%Δ (post vs. pre)		0.57%	0.73%	0.90%	1.42%	1.63%	2.93%
RPText180°/s (%)	Mean	Pre	122.64	124.31	121.87	124.55	125.26	123.22
	SD		9.76	10.81	4.19	7.56	7.08	10.23
	Mean	Post	122.59	124.53	122.13	126.75	128.06	128.14
	SD		9.66	10.74	3.89	6.73	5.97	7.81
	%Δ (post vs. pre)		−0.04%	0.18%	0.21%	1.77%	2.24%	3.99%
RPTflx60°/s (%)	Mean	Pre	83.55	85.15	86.23	84.77	85.37	85.77
	SD		6.78	4.86	3.62	6.51	5.71	5.90
	Mean	Post	83.55	84.95	86.25	86.13	86.22	87.71
	SD		7.12	4.61	3.87	6.92	5.51	6.13
	%Δ (post vs. pre)		0.00%	−0.23%	0.02%	1.60%	1.00%	2.26%
RPTflx180°/s (%)	Mean	Pre	79.00	79.12	78.49	78.69	77.29	78.05
	SD		5.78	4.65	3.41	5.36	5.23	6.05
	Mean	Post	78.82	79.24	78.54	79.97	78.67	80.14
	SD		6.01	4.80	3.89	5.16	5.55	5.84
	%Δ (post vs. pre)		−0.23%	0.15%	0.06%	1.63%	1.79%	2.68%
TPText60°/s (ms)	Mean	Pre	393.00	396.00	385.00	396.00	387.00	396.00
	SD		63.77	64.67	43.01	35.33	41.10	51.03
	Mean	Post	390.00	391.00	380.00	386.00	380.00	383.00
	SD		66.16	67.56	42.16	28.75	49.66	57.35
	%Δ (post vs. pre)		−0.76%	−1.26%	−1.30%	−2.53%	−1.81%	−3.28%
TPText180°/s (ms)	Mean	Pre	265.00	256.00	258.00	268.00	267.00	296.00
	SD		77.92	68.01	51.81	40.77	32.67	44.77
	Mean	Post	260.00	248.00	250.00	255.00	256.00	279.00
	SD		73.78	69.08	55.57	40.62	31.69	44.33
	%Δ (post vs. pre)		−1.89%	−3.13%	−3.10%	−4.85%	−4.12%	−5.74%
TPTflx60°/s (ms)	Mean	Pre	227.00	232.00	227.00	235.00	234.00	236.00
	SD		55.38	50.50	39.45	57.59	50.15	39.77
	Mean	Post	225.00	228.00	222.00	227.00	228.00	226.00
	SD		57.39	50.06	31.90	53.34	60.33	37.17
	%Δ (post vs. pre)		−0.88%	−1.72%	−2.20%	−3.40%	−2.56%	−4.24%
TPTflx180°/s (ms)	Mean	Pre	175.00	174.00	172.00	173.00	168.00	181.00
	SD		32.40	35.65	22.01	30.93	36.75	24.69
	Mean	Post	173.00	171.00	168.00	166.00	163.00	163.00
	SD		33.35	27.26	28.59	25.03	28.30	22.63
	%Δ (post vs. pre)		−1.14%	−1.72%	−2.33%	−4.05%	−2.98%	−9.94%
AvRFDext60°/s (N/s)	Mean	Pre	0.28	0.28	0.28	0.28	0.28	0.29
	SD		0.02	0.02	0.02	0.02	0.01	0.01
	Mean	Post	0.28	0.29	0.29	0.29	0.29	0.31
	SD		0.02	0.02	0.03	0.01	0.01	0.02
	%Δ (post vs. pre)		0.00%	3.57%	3.57%	3.57%	3.57%	6.90%
AvRFDext180°/s (N/s)	Mean	Pre	0.39	0.41	0.40	0.37	0.37	0.33
	SD		0.08	0.11	0.06	0.04	0.03	0.03
	Mean	Post	0.40	0.43	0.42	0.40	0.40	0.36
	SD		0.08	0.11	0.08	0.05	0.03	0.04
	%Δ (post vs. pre)		2.56%	4.88%	5.00%	8.11%	8.11%	9.09%
AvRFDflx60°/s (N/s)	Mean	Pre	0.33	0.32	0.32	0.32	0.32	0.32
	SD		0.05	0.05	0.04	0.06	0.04	0.04
	Mean	Post	0.33	0.33	0.33	0.33	0.34	0.34
	SD		0.05	0.06	0.04	0.06	0.06	0.03
	%Δ (post vs. pre)		0.00%	3.13%	3.13%	3.13%	6.25%	6.25%
AvRFDflx180°/s (N/s)	Mean	Pre	0.39	0.41	0.40	0.40	0.42	0.39
	SD		0.07	0.08	0.04	0.05	0.09	0.06
	Mean	Post	0.40	0.41	0.42	0.42	0.43	0.45
	SD		0.05	0.05	0.05	0.04	0.07	0.07
	%Δ (post vs. pre)		2.56%	0.00%	5.00%	5.00%	2.38%	15.38%
AvPext60°/s (watts)	Mean	Pre	64.87	65.32	64.41	64.93	64.66	68.09
	SD		9.28	9.55	6.25	7.21	8.21	6.81
	Mean	Post	64.94	65.44	64.49	66.86	66.36	70.16
	SD		9.46	9.40	5.95	7.08	8.18	6.51
	%Δ (post vs. pre)		0.11%	0.18%	0.12%	2.97%	2.63%	3.04%
AvPext180°/s (watts)	Mean	Pre	166.68	165.97	167.57	165.63	167.08	165.06
	SD		15.46	18.84	11.47	15.94	12.77	14.06
	Mean	Post	166.33	166.01	167.98	170.17	171.77	171.16
	SD		15.44	18.80	11.22	15.08	13.17	13.04
	%Δ (post vs. pre)		−0.21%	0.02%	0.24%	2.74%	2.81%	3.70%
AvPflx60°/s (watts)	Mean	Pre	54.17	53.16	53.30	54.11	54.01	55.04
	SD		6.04	6.11	3.69	7.52	7.96	5.74
	Mean	Post	54.50	53.62	53.87	55.69	55.24	56.94
	SD		6.22	6.11	3.53	6.05	7.87	5.45
	%Δ (post vs. pre)		0.61%	0.87%	1.07%	2.92%	2.28%	3.45%
AvPflx180°/s (watts)	Mean	Pre	92.69	92.64	93.50	91.05	92.94	94.41
	SD		7.36	8.75	5.32	10.42	8.10	6.44
	Mean	Post	92.75	92.77	93.59	92.84	94.58	96.54
	SD		7.41	8.70	5.21	11.02	7.59	5.95
	%Δ (post vs. pre)		0.06%	0.14%	0.10%	1.97%	1.76%	2.26%
MVICext45° (Nm)	Mean	Pre	106.57	107.73	107.50	108.35	106.51	106.59
	SD		19.13	18.51	15.80	18.63	17.63	13.73
	Mean	Post	106.31	107.72	107.60	109.65	107.76	109.35
	SD		19.20	18.11	16.40	18.53	17.19	13.39
	%Δ (post vs. pre)		−0.24%	−0.01%	0.09%	1.20%	1.17%	2.59%
MVICext60° (Nm)	Mean	Pre	121.01	122.63	123.38	124.08	122.16	121.55
	SD		19.30	17.59	12.20	18.13	17.24	12.04
	Mean	Post	120.55	122.60	123.26	125.58	123.14	125.39
	SD		19.34	17.25	12.31	18.17	17.38	10.51
	%Δ (post vs. pre)		−0.38%	−0.02%	−0.99%	1.21%	0.80%	3.16%
MVICflx45° (Nm)	Mean	Pre	74.53	76.06	75.35	76.38	76.64	79.83
	SD		8.83	6.80	4.13	7.28	8.23	5.47
	Mean	Post	74.51	75.68	75.28	77.13	77.96	81.71
	SD		9.11	6.94	3.97	7.29	8.53	4.93
	%Δ (post vs. pre)		−0.03%	−0.50%	−0.09%	0.98%	1.72%	2.36%
MVICflx60° (Nm)	Mean	Pre	72.61	73.86	72.68	73.96	74.83	75.80
	SD		5.21	4.45	2.77	4.02	4.01	2.80
	Mean	Post	72.42	73.89	72.58	75.54	75.60	77.70
	SD		4.90	3.95	2.68	3.65	3.77	2.57
	%Δ (post vs. pre)		−0.26%	0.04%	−0.14%	2.14%	1.03%	2.51%

CON: control; USRPT: ultra short race pace training; PLA + USRPT: placebo and training; PRO + USRPT: probiotics and training; OMEGA + USRPT: omega-3 and training; PRO + OMEGA + USRPT: probiotics with omega-3 and training; %Δ (post vs. pre): relative change; DHGS: dominant hand grip strength; NDHGS: non-dominant hand grip strength; TMB: throwing medicine ball; SEDH: straight-elbow dead-hang; FEDH: flexed-elbow dead-hang; APT: absolute peak torque; RPT: relative peak torque; TPT: time-to-peak torque; AvRFD: average rate of force development; AvP: average power; MVIC: maximum voluntary isometric contraction; ext: extension; flx: flexion; kg: kilogram; m: meter; Nm: Newton meter; ms: millisecond; N/s: Newtons per second.

**Table 4:** Comparison of the data between pre-test and post-test variables in the six groups.

Variables			CON	USRPT	PLA + USRPT	PRO + USRPT	OMEGA + USRPT	PRO + OMEGA + USRPT
			Post					
DHGS (kg)	MD	Pre	0.90	1.60	1.60	3.80	2.90	4.60
	Sig		0.297	0.067	0.067	0.001	0.001	0.001
	95%CI		−0.81–2.61	−0.11–3.31	−0.11–3.31	2.08–5.51	1.18–4.61	2.88–6.31
NDHGS (kg)	MD	Pre	1.00	0.60	−0.20	2.30	0.30	2.00
	Sig		0.266	0.466	0.807	0.007	0.715	0.018
	95%CI		−0.63–2.63	−1.03–2.23	−1.83–1.43	0.66–3.93	−1.33–1.93	0.36–3.63
TMB (m)	MD	Pre	0.14	0.13	0.10	0.22	0.18	0.23
	Sig		0.114	0.156	0.273	0.018	0.041	0.014
	95%CI		−0.03–0.32	−0.05–0.31	−0.08–0.28	0.03–0.40	0.01–0.36	0.04–0.41
SEDH (kg)	MD	Pre	0.40	1.50	1.50	1.80	1.80	2.30
	Sig		0.678	0.123	0.123	0.065	0.065	0.020
	95%CI		−1.51–2.31	−0.41–3.41	−0.41–3.41	−0.11–3.71	−0.11–3.71	0.38–4.21
FEDH (kg)	MD	Pre	0.70	1.50	1.60	2.00	1.60	3.30
	Sig		0.337	0.043	0.031	0.008	0.031	0.001
	95%CI		−0.74–2.14	0.05–2.94	0.15–3.04	0.55–3.44	0.15–3.04	1.85–4.74
APText60°/s (Nm)	MD	Pre	1.23	2.77	1.90	3.49	3.19	5.50
	Sig		0.101	0.001	0.013	0.001	0.001	0.001
	95%CI		−0.24–2.70	1.29–4.24	0.42–3.37	2.01–4.96	1.71–4.66	4.02–6.97
APText180°/s (Nm)	MD	Pre	−0.25	0.37	0.37	1.97	2.43	3.41
	Sig		0.601	0.440	0.440	0.001	0.001	0.001
	95%CI		−1.20–0.70	−0.58–1.32	−0.58–1.32	1.01–2.92	1.45–3.38	2.45–4.36
APTflx60°/s (Nm)	MD	Pre	0.38	0.52	0.79	2.01	1.52	2.43
	Sig		0.339	0.192	0.060	0.001	0.001	0.001
	95%CI		−0.41–1.17	−0.27–1.31	−0.01–1.58	1.22–2.80	0.73–2.31	1.64–3.22
APTflx180°/s (Nm)	MD	Pre	0.39	0.42	0.46	1.39	1.38	2.06
	Sig		0.366	0.330	0.287	0.002	0.002	0.001
	95%CI		−0.46–1.24	−0.43–1.27	−0.39–1.31	0.53–2.24	0.52–2.23	1.20–2.91
RPText60°/s (%)	MD	Pre	0.77	0.99	1.21	1.95	2.21	3.98
	Sig		0.150	0.066	0.026	0.001	0.001	0.001
	95%CI		−0.28–1.82	−0.06–2.04	0.15–2.26	0.89–3.00	1.15–3.26	2.92–5.03
RPText180°/s (%)	MD	Pre	−0.05	0.22	0.26	2.20	2.80	4.92
	Sig		0.927	0.689	0.636	0.001	0.001	0.001
	95%CI		−1.14–1.04	−0.87–1.31	−0.83–1.35	1.10–3.29	1.70–3.89	3.82–6.01
RPTflx60°/s (%)	MD	Pre	0.00	−0.20	0.02	1.36	0.85	1.94
	Sig		1.000	0.466	0.942	0.001	0.003	0.001
	95%CI		−0.54–0.54	−0.74–0.34	−0.52–0.56	0.81–1.90	0.30–1.39	1.39–2.48
RPTflx180°/s (%)	MD	Pre	−0.18	0.12	0.05	1.28	1.38	2.09
	Sig		0.358	0.539	0.798	0.001	0.001	0.001
	95%CI		−0.56–0.20	−0.50–0.26	−0.43–0.33	0.89–1.66	0.99–1.76	1.70–2.47
TPText60°/s (ms)	MD	Pre	−3.00	−5.00	−5.00	−10.00	−7.00	−13.00
	Sig		0.614	0.402	0.402	0.097	0.242	0.032
	95%CI		−14.87–8.87	−16.87–6.87	−16.87–6.87	−21.87–1.87	−18.87–4.87	−24.87–−1.13
TPText180°/s (ms)	MD	Pre	−5.00	−8.00	−8.00	−13.00	−11.00	−17.00
	Sig		0.270	0.080	0.080	0.005	0.018	0.001
	95%CI		−13.99–3.99	−16.99–0.99	−16.99–0.99	−21.99–−4.00	−19.99–−2.00	−25.99–−8.00
TPTflx60°/s (ms)	MD	Pre	−2.00	−4.00	−5.00	−8.00	−6.00	−10.00
	Sig		0.668	0.393	0.287	0.091	0.202	0.036
	95%CI		−11.31–7.31	−13.31–5.31	−14.31–4.31	−17.31–1.31	−15.31–3.31	−19.31–−0.68
TPTflx180°/s (ms)	MD	Pre	−2.00	−3.00	−4.00	−7.00	−5.00	−18.00
	Sig		0.694	0.556	0.433	0.172	0.328	0.001
	95%CI		−12.14–8.14	−13.14–7.14	−14.14–6.14	−17.14–3.14	−15.14–5.14	−28.14–−7.85
AvRFDext60°/s (N/s)	MD	Pre	0.00	0.01	0.01	0.01	0.01	0.02
	Sig		0.347	0.042	0.063	0.010	0.002	0.001
	95%CI		−0.01–0.01	0.01–0.02	−0.01–0.02	0.01–0.02	0.01–0.02	0.01–0.03
AvRFDext180°/s (N/s)	MD	Pre	0.00	0.01	0.01	0.02	0.02	0.03
	Sig		0.520	0.043	0.017	0.001	0.001	0.001
	95%CI		−0.01–0.02	0.01–0.03	0.01–0.03	0.01–0.04	0.01–0.04	0.01–0.04
AvRFDflx60°/s (N/s)	MD	Pre	0.00	0.00	0.00	0.01	0.02	0.02
	Sig		0.507	0.235	0.354	0.020	0.005	0.002
	95%CI		−0.01–0.02	−0.01–0.02	−0.01–0.02	0.01–0.03	0.01–0.03	0.01–0.04
AvRFDflx180°/s (N/s)	MD	Pre	0.00	0.00	0.01	0.02	0.01	0.05
	Sig		0.719	0.829	0.252	0.135	0.252	0.001
	95%CI		−0.02–0.03	−0.02–0.03	−0.01–0.04	−0.01–0.04	−0.01–0.04	0.02–0.08
AvPext60°/s (watts)	MD	Pre	0.07	0.12	0.08	1.93	1.73	2.07
	Sig		0.758	0.598	0.725	0.001	0.001	0.001
	95%CI		−0.38–0.52	−0.33–0.57	−0.37–0.53	1.47–2.38	1.27–2.18	1.61–2.52
AvPext180°/s (watts)	MD	Pre	−0.35	0.04	0.41	4.54	4.69	6.10
	Sig		0.485	0.936	0.414	0.001	0.001	0.001
	95%CI		−1.34–0.64	−0.95–1.03	−0.58–1.40	3.54–5.53	3.69–5.68	5.10–7.09
AvPflx60°/s (watts)	MD	Pre	0.33	0.46	0.57	1.58	1.23	1.90
	Sig		0.313	0.161	0.084	0.001	0.001	0.001
	95%CI		−0.32–0.98	−0.19–1.11	−0.08–1.22	0.93–2.23	0.58–1.88	1.25–2.55
AvPflx180°/s (watts)	MD	Pre	0.06	0.13	0.09	1.79	1.64	2.13
	Sig		0.829	0.639	0.745	0.001	0.001	0.001
	95%CI		−0.49–0.61	−0.42–0.68	−0.46–0.64	1.23–2.34	1.08–2.19	1.57–2.68
MVICext45° (Nm)	MD	Pre	−0.26	−0.01	0.10	1.30	1.25	2.76
	Sig		0.256	0.965	0.660	0.001	0.001	0.001
	95%CI		−0.71–0.19	−0.46–0.44	−0.35–0.55	0.84–1.75	0.79–1.70	2.30–3.21
MVICext60° (Nm)	MD	Pre	−0.46	−0.03	−0.12	1.50	0.98	3.84
	Sig		0.200	0.933	0.736	0.001	0.008	0.001
	95%CI		−1.17–0.25	−0.74–0.68	−0.83–0.59	0.79–2.21	0.27–1.69	3.31–4.55
MVICflx45° (Nm)	MD	Pre	−0.02	−0.03	−0.07	0.75	1.32	1.88
	Sig		0.928	0.090	0.752	0.001	0.001	0.001
	95%CI		−0.46–0.42	−0.82–0.06	−0.51–0.37	0.30–1.19	0.87–1.76	1.43–2.32
MVICflx60° (Nm)	MD	Pre	−0.19	0.03	−0.10	1.58	0.77	1.90
	Sig		0.382	0.890	0.645	0.001	0.001	0.001
	95%CI		−0.662–0.24	−0.40–0.46	−0.53–0.33	1.14–2.01	0.33–1.20	1.46–2.33

CON: control; USRPT: training; PLA + USRPT: placebo and training; PRO + USRPT: probiotics and training; OMEGA + USRPT: omega-3 and training; PRO + OMEGA + USRPT: probiotics with omega-3 and training; DHGS: dominant handgrip strength; NDHGS: non-dominant handgrip strength; TMB: throwing medicine ball; SEDH: straight-elbow dead-hang; FEDH: flexed-elbow dead-hang; APT: absolute peak torque; RPT: relative peak torque; TPT: time-to-peak torque; AvRFD: average rate of force development; AvP: average power; MVIC: maximum voluntary isometric contraction; ext: extension; flx: flexion; kg: kilogram; m: meter; Nm: Newton meter; ms: millisecond; N/s: Newtons per second; MD: mean difference; CI: confidence interval; Sig: Significant difference.

## Data Availability

The data presented in this study are available on request from the corresponding author due to due to privacy or ethical restrictions.
